# New setting of neurally adjusted ventilatory assist during mask noninvasive ventilation

**DOI:** 10.1186/cc13458

**Published:** 2014-03-17

**Authors:** F Longhini, C Pan, G Cammarota, R Vaschetto, J Xie, L Liu, Y Yian, F Della Corte, P Navalesi, H Qiu

**Affiliations:** 1 Universita del Piemonte Orientale, Novara, Italy; 2 Southeast University, Nanjing, China

## Introduction

Noninvasive ventilation through a mask is commonly applied in pressure support ventilation (nPSV). Recent studies showed that noninvasive neurally adjusted ventilatory assist (nNAVA) improves patient-ventilator interaction and synchrony. More recently we described a new setting for nNAVA (nNAVA15) able to reduce the peak of electrical activity of the diaphragm (EAdipeak) and dyspnea (assessed by a visual analogue scale, VASd), compared with both nPSV and nNAVA, in patients undergoing NIV through a helmet, by improving the rate pressurization. We therefore designed a physiological study to evaluate and compare the effects of nNAVA15 with nPSV and nNAVA on VASd, EAdipeak, pressurization rate and arterial blood gases (ABGs).

## Methods

Fourteen patients undergoing noninvasive ventilation because of acute respiratory failure underwent three randomized 30-minute trials: nPSV (inspiratory support above positive end- expiratory pressure (PEEP) ≥8 cmH_2_O, fastest rate of pressurization); nNAVA (NAVA level to achieve a comparable EAdipeak as during nPSV); nNAVA15 (NAVA level at 15 cmH_2_O/jV and the maximum inspiratory airway pressure (Paw) set at the value corresponding to PEEP + inspiratory support during nPSV). Oxygen inspiratory fraction and PEEP remained unmodified throughout the study period. The last minute of each trial was analyzed. Paw-time products of the initial 200 ms from the onset of ventilator pressurization (PTP200), of the initial 300 and 500 ms from the onset of the EAdi swing indexed to the ideal PTP (PTP300i and PTP500i, respectively), and of the triggering area (PTPt) were computed. ABGs and VASd were assessed at the end of each trial.

## Results

nNAVA15 reduced VASd (3.0 (2.0; 3.0)), compared with both nPSV (5.0 (4.0; 5.2)) and nNAVA (4.0 (3.0; 5.0)) (*P *< 0.001), without affecting ABGs and EAdipeak. nNAVA15 improved PTP300i and PTP500i (42% (32.5; 46.5) and 63% (54; 68)%, respectively) compared with nPSV (25 (4; 33)% and 44 (23; 52)%, *P *< 0.001) and nNAVA (25 (20; 34)% and 46 (33; 57)%, *P *< 0.001). PTP200 was lower in nNAVA (62 (46; 82) cmH_2_O*second) with respect to both nPSV (87 (77; 112) cmH_2_O*second) and nNAVA15 (85 (70; 127) cmH_2_O*second) (*P *= 0.001). PTPt was significantly improved by both nNAVA (-00.9 (-3.2; -0.2) cmH_2_O*second) and nNAVA15 (-0.6 (-2.3; -0.2) cmH_2_O*second) as opposed to nPSV (-9.4 (-12.3; -5.9) cmH_2_O*second, *P *< 0.001).

## Conclusion

Compared with nPSV and nNAVA, nNAVA15 through a mask reduces VASd, assuring an optimal pressurization rate and triggering performance, without affecting the breathing pattern, neural drive and ABGs.

